# The Effects on Saturated Fat Purchases of Providing Internet Shoppers with Purchase- Specific Dietary Advice: A Randomised Trial

**DOI:** 10.1371/journal.pctr.0010022

**Published:** 2006-09-22

**Authors:** Amy Huang, Federica Barzi, Rachel Huxley, Gareth Denyer, Beth Rohrlach, Kathy Jayne, Bruce Neal

**Affiliations:** 1 George Institute for International Health, University of Sydney, Sydney, New South Wales, Australia; 2 Department of Biochemistry, University of Sydney, Sydney, New South Wales, Australia

## Abstract

**Objectives:**

The supermarket industry now services many customers through online food shopping over the Internet. The Internet shopping process offers a novel opportunity for the modification of dietary patterns. The aim of this study was to evaluate the effects on consumers' purchases of saturated fat of a fully automated computerised system that provided real-time advice tailored to the consumers' specific purchases recommending foods lower in saturated fat.

**Design:**

This study was a blinded, randomised controlled trial.

**Setting:**

The study was conducted in Sydney, New South Wales, Australia.

**Participants:**

The participants were consumers using a commercial online Internet shopping site between February and June 2004.

**Interventions:**

Individuals assigned to intervention received fully automated advice that recommended specific switches from selected products higher in saturated fat to alternate similar products lower in saturated fat. Participants assigned to control received general non-specific advice about how to eat a diet lower in saturated fat.

**Outcome Measures:**

The outcome measure was the difference in saturated fat (grams per 100 g of food) in shopping baskets between the intervention and control groups.

**Results:**

There were 497 randomised participants, mean age 40 y, each shopping for an average of about three people. The amount of saturated fat in the foods purchased by the intervention group was 0.66% lower (95% confidence interval 0.48–0.84, *p* < 0.001) than in the control group. The effects of the intervention were sustained over consecutive shopping episodes, and there was no difference in the average cost of the food bought by each group.

**Conclusions:**

Fully automated, purchase-specific dietary advice offered to customers during Internet shopping can bring about changes in food purchasing habits that are likely to have significant public health implications. Because implementation is simple to initiate and maintain, this strategy would likely be highly cost-effective.

## Editorial Commentary

**Background:** Current evidence from randomized trials and observational studies indicates that people in the developed world should try to lower their intake of saturated fat, in order to reduce the risk of cardiovascular diseases such as heart attacks and strokes. However, achieving this aim in the population is not straightforward, partly because of the high cost of personalized interventions and, often, their limited success. One possible way of delivering dietary advice that might help people to make healthier food choices is through Internet shopping systems. In the trial reported here, which was carried out in Sydney, New South Wales, Australia, people using an Internet food shopping system and who were about to complete their purchases were offered the chance to participate in the trial. If they agreed, participants were immediately randomized to receive either general dietary advice (the trial's control) or tailored advice delivered by a computer program that prompted shoppers to replace specific items in their shopping basket with alternatives lower in saturated fat. The main outcome the trial researchers were interested in was the percent of saturated fat in the food bought, compared between the two arms of the trial. The researchers also looked at the cost per gram of the food that was bought.

**What this trial shows:** The researchers recruited 456 participants into the trial. They found that the participants who received tailored advice bought foods containing less saturated fat, on average, than the participants who received only general advice. The difference between the two groups amounted to around 10% of the average quantity of saturated fat in food chosen before the advice was offered, and this difference was statistically significant. Finally, there was no significant difference in the average cost of food between the two arms of the trial.

**Strengths and limitations:** The trial recruited enough participants to evaluate whether this intervention had an effect on the main outcomes, and the randomization procedures used and outcomes measured were appropriate. A potential strength of this study is that it evaluates a low-cost technology that might be useful alongside other strategies for reducing saturated fat intake. However, the trial was conducted among people who are technically aware, and may not therefore represent the general population as a whole. In the trial, the outcomes measured were limited to information about the foods bought, and the effects on cardiovascular risk or health outcomes were not examined.

**Contribution to the evidence:** Systematic reviews of existing randomized trials already show that offering dietary advice can bring about improvements in diet (such as saturated fat intake) and cardiovascular risk. This trial adds to that evidence by showing that tailored advice such as can be delivered via an Internet shopping system is likely to be a useful method of encouraging healthier food purchases.

## INTRODUCTION

Fats consumed in food are an important cause of dyslipidaemia and cardiovascular disease [1–3]. There is clear evidence from randomised controlled trials that significant improvements in blood lipid parameters can be achieved by modifying dietary fat intake [4], and for some time there has been a consensus that reduced consumption of saturated fat should be a goal for the populations of many higher-income countries [5,6]. A broad range of strategies for the modification of dietary fat consumption have been designed and implemented with varying degrees of success [7–10]. Key factors for efficacy of dietary interventions appear to be the intensity of advice offered and the tailoring of suggestions to individual's specific purchases [11–13]. Unfortunately, the high cost associated with providing such advice has precluded the widespread implementation of some of the most effective programs.

Over the last decade many supermarkets have introduced online food purchasing facilities. The computer interface offers a unique new opportunity to deliver automated, purchase-specific dietary advice to large numbers of individuals at low cost [14–16]. We report here the results of a randomised trial designed to determine whether the provision of such advice could result in reductions in the amount of saturated fat in foods purchased by consumers using a commercial Internet shopping facility.

## METHODS

The Dietary Intervention in e-Shopping Trial was a blinded, randomised controlled trial conducted in collaboration with an established supermarket providing an Internet shopping service in Sydney, New South Wales, Australia. The software program was developed in conjunction with Simbient Solutions (North Sydney, New South Wales, Australia). Recruitment and follow-up was done between February and June 2004. The study was approved by the Ethics Committee of the University of Sydney, and all participants provided informed consent online.

### Participants

All customers of the online supermarket service that made any purchase after the trial commencement date (February 2004) were offered the opportunity to participate until recruitment was closed (April 2004). Recruitment was via an online electronic pop-up message. The opportunity to participate was offered after the selection of items for purchase had been completed, just prior to online checkout. Interested participants were first asked to read an online participant information sheet and then to complete an online consent form. Once this was done, a simple baseline data collection form comprising questions about basic demographics, shopping habits, and selected medical conditions was provided for completion. The entire trial enrolment process was estimated to take only 5–10 min.

### Interventions

A comprehensive list of all food products purchased online listed in order of frequency of purchase was obtained from the commercial partner operating the Internet shopping service. In total, 383 commonly purchased food items that contained 1% or more saturated fat (range 1% to 92%) were selected, and a suitable lower-fat alternate was identified for each. There were 524 discrete foods identified in this process. Food items that were not prepackaged, such as some meat products, were excluded from our study since reliable information about saturated fat content was not easily available.

#### Intervention.

Participants assigned to intervention received advice tailored to the food items they had selected for purchase (Figure S1). This was done automatically by a computer program that compared the food items selected for purchase against the list of identified items containing 1 g or more of saturated fat per 100 g of food. For each such item that was identified the participant was presented with the opportunity to either retain it or to swap it for the alternate lower in saturated fat. A simple side-by-side on-screen presentation of the original item and the suggested alternate was used. Once the participants had made their decisions, they were returned to the usual online checkout process.

#### Control.

Participants assigned to control received general non-specific advice about how to choose a diet lower in saturated fat (Figure S2). This advice was presented in the form of a static Web page and was based on information provided on the Web site of the National Heart Foundation of Australia. Once participants had read the advice they were prompted to make any changes to their planned purchases on the basis of the information they had read and then returned to the usual online checkout process.

### Objectives

The objective of the study was to determine whether the provision of fully automated and purchase-specific dietary advice could produce reductions in the amount of saturated fat in foods purchased by consumers using a commercial Internet shopping facility.

### Outcomes

The primary outcome was the mean percent of saturated fat in the purchased items that were among the 524 foods studied. The secondary outcome was the mean cost per 100 g of these same items. Participants were offered the same form of advice each time that they used the online supermarket during the 5-mo recruitment and follow-up period (February to June 2004). On each occasion after the first they could choose either to receive the advice or simply to proceed straight to the online checkout. On every occasion the contents of the online shopping basket were recorded before and after the advice (or the opportunity to receive the advice) was offered.

### Sample Size

A sample size of 500 participants was selected to provide at least 90% power with α = 0.05 to detect a 2% or greater difference in the mean saturated fat content (in grams per 100 g of food) of purchased foods between randomised groups. The power calculation was based on assumptions derived from a 1986 trial with broadly similar goals that used an in-store computer terminal [17].

### Randomisation and Blinding

Once the recruitment process was completed, participants were assigned to intervention or control at random by a central computerised process with minimisation by age, sex, and number of individuals the food was being purchased for. Randomisation was done in real time as each participant was recruited using a computer program designed and tested by statisticians at the George Institute for International Health in Sydney, New South Wales, Australia. Once randomised participants were immediately provided with the form of advice consistent with their randomised assignment. The participants were unaware of treatment allocation, and the principal investigator performed unblinding of the treatment allocation once the study follow-up had ended.

### Statistical Methods

The content of saturated fat in each shopping basket (separately for the shopping baskets before and after advice was provided) was calculated for each participant by dividing the sum in grams of all the saturated fat by the sum in grams of all the food, for the 524 foods studied. Primary analyses were based on the first shopping episode for each individual that included one of the 524 foods in either a “before advice” or “after advice” shopping basket. For the 41 (8%) individuals that never purchased one of the 524 foods, the saturated fat content before and after was imputed using the method of multiple imputation [18]. The difference in mean saturated fat content of the “after” compared to the “before” shopping baskets was calculated separately for intervention and control and compared between groups using linear regression models. The linear regression models included the amount of saturated fat selected prior to advice being offered, since there was by chance a small imbalance in this value between the randomised groups (control 5.9%, intervention 7.1%) [19]. An arcsine square-root transformation of the saturated fat content (in grams per 100 g of food) was used since the data were skewed, with the results transformed back after analysis. A series of sensitivity analyses were done to test the stability of the main result: the effect estimate was first recalculated excluding participants with missing values for the primary outcome, and then recalculated with these participants included but having the change in saturated fat set as zero. Second, the results were calculated without adjustment for saturated fat, and separate linear regression models were fitted with adjustment for a series of possible confounders including age, gender, body mass index, and a variable indicating whether the participant usually tried to buy low-fat food before being recruited on the trial. Subgroup analyses were also done to explore the constancy of the intervention across the participant population by including in the linear models a randomised group by covariate interaction term. The effects of repeated presentation of the advice were determined by using linear mixed effects models [20]. All the statistical analyses were done using SAS/STAT version 8.2 (SAS Institute, Cary, North Carolina, United States).

## RESULTS

### Participants

**Figure 1:**
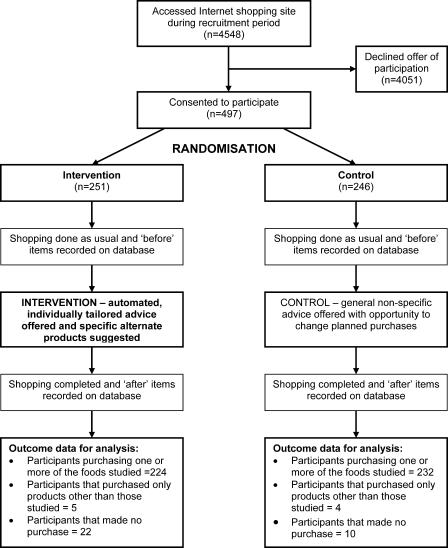
Flow Chart of Study

A total of 4,548 individuals were offered participation, and 497 were randomised (251 intervention and 246 control) (Figure 1). Of these, 456 (224 intervention and 232 control) completed at least one episode of shopping that included one or more of the 524 foods studied. Median follow-up time completed by the end of the study in June 2004 was 35 d, and the median number of shopping episodes done by participants was three (range 1–20). The baseline characteristics documented on the questionnaire were balanced between randomised groups, with a mean participant age of 40 y and a proportion female of 88% (Table 1).

**Table 1:**
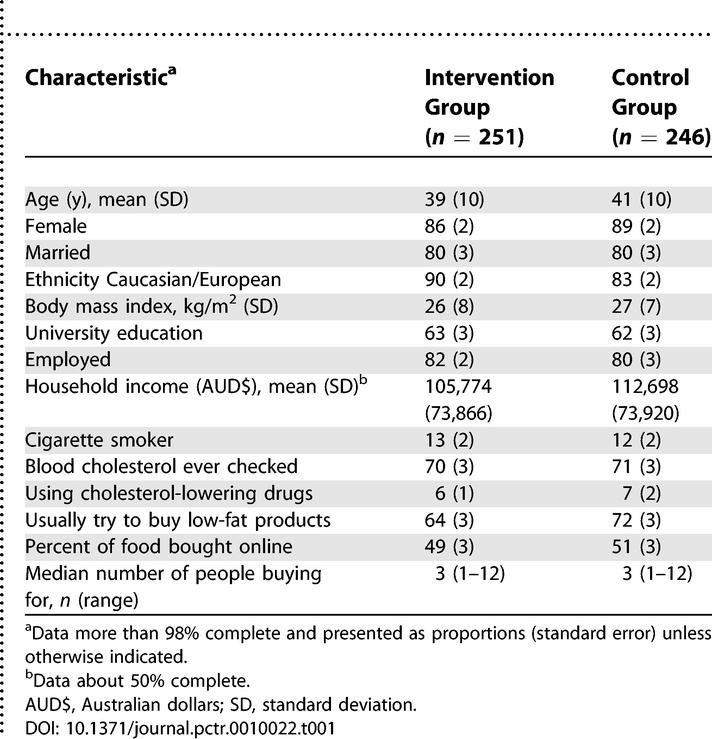
Baseline Characteristics

### Outcomes and Estimation

**Figure 2:**
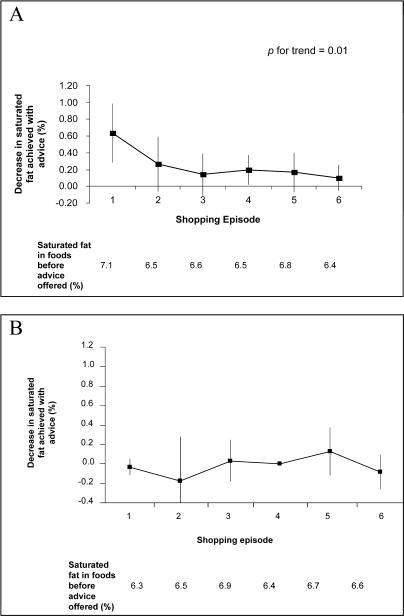
Effects of Repeated Advice in the Intervention (*n =* 115) and Control Group (*n =* 121) Squares are placed at the point estimate of the effect observed in the intervention (A) and control (B) groups, and the vertical lines extend to the 95% confidence intervals around the estimate. The *p*-value for trend in the intervention group indicates a progressive decrease in effect size with repeated shopping episodes. There was no significant decrease in saturated fat at any time point in the control group, nor any trend over time (all *p* > 0.09).

For the first occasion on which advice was offered, the amount of saturated fat in the food purchased by the intervention group after advice was a mean of 0.66% (0.48–0.84, *p <* 0.001) lower than in the corresponding foods purchased by the control group (Table 2), which is equivalent to an approximate 10% reduction in saturated fat content of foods purchased (Figure 2). This difference resulted from a decrease in the mean saturated fat content in the foods purchased following the advice offered to the intervention group of 0.77% (0.60–0.94, *p <* 0.001), with no corresponding decrease in the control group 0.04% (0.00–0.08, *p =* 0.07). The effect estimate for the primary outcome was 0.62% (0.46–0.79, *p <* 0.001) if analysis was restricted to only the 456 individuals that selected one of the 524 foods studied and was 0.58 (0.39–0.77, *p <* 0.001) if the change in saturated fat was set to zero for those individuals that did not select one of the foods. The subgroup analyses provided some evidence that the intervention had greater effects among individuals with higher body mass index and among people above 40 y of age (for both, homogeneity *p* < 0.03) (Table 3). There was no baseline variable that substantively altered the main result as a consequence of its inclusion or exclusion as a covariate.

**Table 2:**
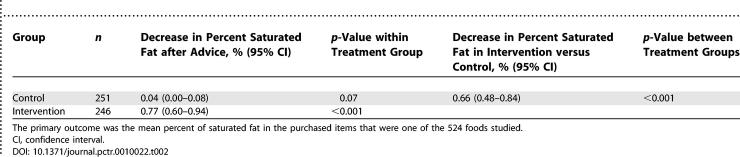
Effects of Intervention on Primary Outcome

**Table 3:**
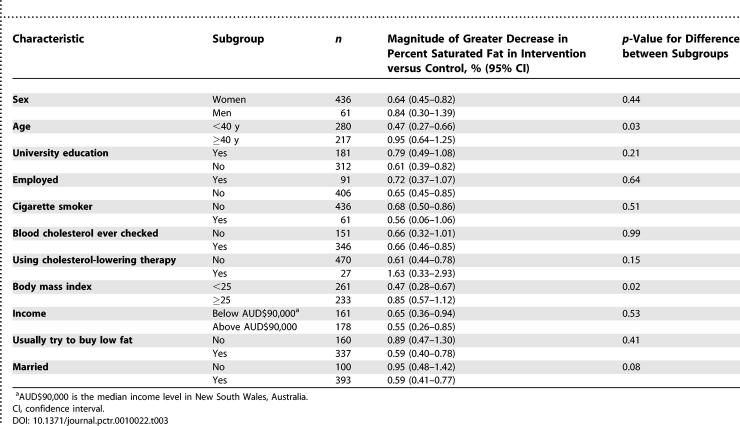
Effects of Intervention on Primary Outcome in Main Participant Subgroups

A secondary outcome of the study was to assess the impact of the intervention on the cost of foods purchased online. The mean cost per 100 g of the foods purchased by the intervention group was not different from that in the control group (intervention AUD$0.63 [0.58–0.68]/100 g versus control AUD$0.62 [0.58–0.67]/100 g, *p =* 0.19). The foods higher in saturated fat that were most commonly present in the shopping basket prior to advice being offered but absent after the advice had been offered were higher-fat dairy products. Lower-fat dairy products were the items most frequently added to the shopping basket after advice was provided.

### Ancillary Analyses

The effects of the intervention over repeated episodes of shopping were explored amongst the 115 participants that completed six shopping episodes during the study. These analyses demonstrated that for the intervention group the magnitude of the reduction in saturated fat achieved was greater in earlier compared to later shopping episodes (for trend, *p* = 0.01) (Figure 2) and showed that there was no effect of the control condition on saturated fat during any shopping episode (*p* > 0.09 for all six shopping episodes in the control group).

## DISCUSSION

### Interpretation

This study clearly demonstrates that it is possible to significantly influence consumers' Internet shopping habits with a simple automated computer algorithm that provides advice tailored to planned food purchases at the point of sale. Changes in saturated fat purchasing habits of the type achieved in this trial are likely to produce long-term reductions in cardiovascular disease mediated through favourable changes to blood lipid profiles [4,9]. Furthermore, the computer algorithm was designed and implemented at a total cost of just a few tens of thousands of dollars and required little maintenance during the 5-mo study period. While periodic updating of the lists of products defined as high in saturated fat and their alternates would be required if implementation were longer term, the resource implications would be small. With anticipated growth in online shopping in Australia [21] and other developed countries, the type of approach proven here could be a cost-effective, long-term, non-drug strategy for the prevention of cardiovascular disease.

### Generalisability

The Dietary Intervention in e-Shopping Trial was a large randomised controlled trial, which should have ensured that both systematic and random errors were minimised and that the study findings are both reliable and precise. As only about one in ten of the consumers that accessed the Internet shopping service during the study period actually participated in the trial, the study participants are clearly not a representative sample of the general population and may not even be representative of those that do Internet shopping. However, the analyses presented here demonstrate that the intervention was similarly effective in most of the subgroups studied, suggesting that the tool is potentially beneficial to a broad range of individuals that utilise Internet shopping services. Furthermore, significantly greater uptake might be expected in other circumstances. Specifically, in a non-trial setting, consumers would not be required to read a lengthy participant information sheet, enter personal details on an online consent form, or complete a baseline questionnaire. In conjunction with marketing activities suitable for a health promotion campaign but inappropriate for a research project, these changes would likely greatly increase the proportion of individuals making use of the service.

### Overall Evidence

The absolute cardiovascular risk of most of our study participants would have been low, and the immediate effects of this intervention on cardiovascular events would be limited. However, if a longer-term view is taken it is clear that the potential of this strategy is substantial. First, participants using the system appeared able to learn to select foods, particularly dairy items, that were lower in saturated fat after receiving advice on just one occasion; retention was probably aided by the fact that computer shopping programs store prior shopping lists for future use. The high fat content of dairy items, such as full-fat milk and cheese, combined with a wide range of lower-fat substitutes that are currently available, would partly explain why these food items were particularly popular switch-over items with consumers in this current study. By contrast, in the Women's Health Initiative trial [22], reduction in fat intake primarily came from changes in meats and desserts. We postulate that this difference is likely due to the fact that items such as milk and cheese are more likely to be purchased using the Internet shopping facility, whereas more expensive food items, such as meat and desserts, are purchased using more conventional means. The lesser effect of the intervention in later shopping episodes may have been because from the second shopping episode onwards the intervention group appeared to have learned to select foods with a lower percentage of saturated fat before receiving the advice. Second, the decisions to select items lower in saturated fat influenced shopping purchases not just for the index case but also for an average of two other individuals that the index case was shopping for. Third, half of all the food purchased by the participants for their household was exposed to review by our system. Fourth, while the apparently greater effects of the intervention among those with higher body mass index and older age could be chance findings, these results may also indicate that the intervention is of particular use among a group that is at increased risk of cardiovascular disease. Finally, changing dietary patterns of the currently young and healthy should translate into substantial long-term benefits with increasing age [7].

The tool we developed specifically targeted purchases of saturated fat. It is easy to imagine an adaptation of the system that could provide advice about salt intake or more sophisticated versions able to address multiple nutritional determinants of health simultaneously and provide broader “heart healthy” dietary advice. However, as Internet supermarket shopping currently accounts for only a small proportion of all groceries consumed, such a system should be used in conjunction with more conventional methods for delivering dietary advice. It is also possible to conceive of interfaces able to provide advice to consumers with specific disease states such as diabetes, high blood pressure, or high cholesterol. With the concept of automated consumer-tailored computer advice now of proven benefit in a commercial setting, the challenge will be to see the results translated into practice. This will require imaginative approaches developed in collaboration with public health advocacy groups, regulatory bodies, and the food retail industry.

## SUPPORTING INFORMATION

CONSORT Checklist Click here for additional data file. (50 KB DOC)

Trial Protocol Click here for additional data file. (309 KB DOC)

Figure S1 Screenshot for the Intervention Group (58 KB DOC)

Figure S2 Screenshot for the Control Group (155 KB DOC)

## References

[pctr-0010022-b001] Hu F, Stampfer M, Manson J, Rimm E, Colditz G (1997). Dietary fat intake and the risk of coronary heart disease in women. N Engl J Med.

[pctr-0010022-b002] Bilenko N, Fraser D, Vardi H, Shai I, Shahar D (2005). Mediterranean diet and cardiovascular diseases in an Israeli population. Prev Med.

[pctr-0010022-b003] Laaksonen D, Nyyssonen K, Niskanen L, Rissanen T, Salonen J (2005). Prediction of cardiovascular mortality in middle-aged men by dietary and serum linoleic and polyunsaturated fatty acids. Arch Intern Med.

[pctr-0010022-b004] Clarke R, Frost C, Collins R, Appleby P, Peto R (1997). Dietary lipids and blood cholesterol: Quantitative meta-analysis of metabolic ward studies. BMJ.

[pctr-0010022-b005] Dietary Guidelines Advisory Committee (1995). Report of the Dietary Guidelines Advisory Committee on the dietary guidelines for Americans, 1995.

[pctr-0010022-b006] Krauss R, Eckel RH, Howard B, Appel LJ, Daniels SR (2000). AHA dietary guidelines: Revision 2000: A statement for healthcare professionals from the Nutrition Committee of the American Heart Association. Circulation.

[pctr-0010022-b007] Tang J, Armitage J, Lancaster T, Silagy C, Fowler G (1998). Systematic review of dietary intervention trials to lower blood total cholesterol on free-living subjects. BMJ.

[pctr-0010022-b008] Brunner E, White I, Thorogood M, Bristow A, Curle D (1997). Can dietary interventions change diet and cardiovascular risk factors? A meta-analysis of randomized controlled trials. Am J Public Health.

[pctr-0010022-b009] Hooper L, Summerbell C, Higgins J, Thompson R, Capps N (2001). Dietary fat intake and prevention of cardiovascular disease: Systematic review. BMJ.

[pctr-0010022-b010] Puska P (1992). The North Karelia Project: Nearly 20 years of successful prevention of CVD in Finland. Hygie.

[pctr-0010022-b011] De Bourdeaudhuij I, Brug J (2000). Tailoring dietary feedback to reduce fat intake: An intervention at the family level. Health Educ Res.

[pctr-0010022-b012] Campbell M, DeVellis B, Strecher V, Ammerman A, DeVellis R (1994). Improving dietary behavior: The effectiveness of tailored messages in primary care settings. Am J Public Health.

[pctr-0010022-b013] Windhauser M, Evans M, McCullough M, Swain JF, Lin PH (1999). Dietary adherence in the Dietary Approaches to Stop Hypertension trial. DASH Collaborative Research Group. J Am Diet Assoc.

[pctr-0010022-b014] Tate DF, Wing RR, Winett RA (2001). Using Internet technology to deliver a behavioral weight loss program. JAMA.

[pctr-0010022-b015] Tate D, Wing R, Jackvony E (2003). Effect of internet behavioral counseling on weight loss in adults at risk for type 2 diabetes. JAMA.

[pctr-0010022-b016] Oenema A, Brug J, Lechner L (2001). Web-based tailored nutrition education: Results of a randomized controlled trial. Health Educ Res.

[pctr-0010022-b017] Winett RA, Moore JF, Wagner JL, Hite LA, Leahy (1991). Altering shoppers' supermarket purchases to fit nutritional guidelines: An interactive information system. J Appl Behav Anal.

[pctr-0010022-b018] Schafer J (1997). Analysis of incomplete multivariate data.

[pctr-0010022-b019] Woodward M (1999). Epidemiology: Study design and data analysis.

[pctr-0010022-b020] Verbeke G, Molenberghs G (2000). Linear mixed models for longitudinal data.

[pctr-0010022-b021] Australian Bureau of Statistics (2000). Use of the Internet by householders.

[pctr-0010022-b022] Patterson RE, Kristal A, Rodabough R, Caan B, Lillington L (2003). Changes in food sources of dietary fat in response to an intensive low-fat dietary intervention: Early results from the Women's Health Initiative. J Am Diet Assoc.

