# Supplementary material for: The Effects on Saturated Fat Purchases of Providing Internet Shoppers with Purchase- Specific Dietary Advice: A Randomised Trial
Source: PLoS Clin Trials. 2006 Sep 22;1(5):e22. doi: 10.1371/journal.pctr.0010022 (PMC1574360; doi:10.1371/journal.pctr.0010022)
Supplement: Trial Protocol [file pctr.0010022.sd002.doc]

**The Dietary Intervention**

**in *e*-shopping Trial**

# PROTOCOL

## AIMS

The primary aim of this randomised controlled trial is to investigate whether tailored dietary advice, delivered over the Internet during Internet shopping, is an effective means of achieving long-term modification of online food purchasing habits related to saturated fat. More specifically, the primary hypothesis is:

- That among users of an online grocery shopping facility, compared to control, provision of tailored dietary advice targeting saturated fat, will result in the purchase of foods with a lower average saturated fat content over a 6-month follow-up period.To determine the effects of real-time, tailored dietary advice, on the amount of saturated fat in purchases made by individuals using an Internet grocery shopping facility.

### EXPERIMENTAL DESIGN

- The Dietary Intervention in ‘e’-shopping Trial (DI*e*T) is a randomised controlled trial that will be conducted among 500 individuals that regularly use an established online grocery shopping facility. The trial will be conducted in collaboration with *shopfast.com* that already offers an online grocery shopping service.

### PARTICIPANTS & RECRUITMENT

- Consecutive individuals using the online shopping service will be offered the opportunity to participate in the trial until the study recruitment target of 500 is achieved.
- Individuals will be eligible to enroll in the study only once, and must be planning to use the online shopping facility at least once more within the following 6-month period.
- The option to participate will be given during a usual visit to the online shopping Website - after the items for purchase have been selected (‘baseline shopping list’) and just prior to entering the online ‘checkout’ process, a short on screen message will provide brief information about the study. Shoppers will be given the option either to continue through to the ‘checkout’ as usual, or to consider participating in the study.
- Participants that indicate interest in the study will immediately be provided with detailed information about the study while online and will be asked to provide informed consent to participate.
- Once consent is provided, simple baseline information will be collected by means of an online questionnaire, followed by immediate randomisation to either intervention or control.

**EXCLUSION CRITERIA**

- Individuals who do not intend to use the online shopping facility again within the proceeding 6 months will be excluded from the study. This will be determined by the answer to question 1 in the online questionnaire.
- Any others??

### RANDOMISATION, INTERVENTION AND CONTROL

Randomisation will be done by a central computer using minimization to achieve balance for key factors that might influence the response to the intervention (including: age, gender, baseline percentage of saturated fat in shopping list, number of individuals in household). The two groups will be:

1. Intervention group: individuals assigned to the intervention group will receive immediate, highly tailored advice about the items in the ‘baseline shopping list’ with the aim of reducing the amount of saturated fat purchased. This will be achieved by using a computer algorithm to identify the products that are high in saturated fat and then offering similar, but lower fat, alternatives. Summary information about the impact of selecting these alternatives on the saturated fat content of the shopping list will be provided and the participant will then be free to either purchase the alternatives or retain the original selections. They will then proceed with their modified shopping list (‘first follow-up shopping list’) and complete online ‘checkout’ as usual.
2. Control group: individuals assigned to the control group will receive immediate, general advice about how to choose a diet low in saturated fat and the health advantages this may confer. This information will be delivered via a traditional, static Web page. They will then proceed to online ‘checkout’ as usual.

All advice provided to the intervention and control groups will be in line with accepted Australian Heart Foundation recommendations, the difference between the groups being in the tailoring of the information provided.

### DATA COLLECTION AND FOLLOW-UP

For each randomised participant, follow-up will comprise one or more post-randomisation visits to the online shopping site during the six-month period after randomisation. At the baseline visit a unique study identification number will be assigned and the following information will be collected and stored in the database:

- Participant demographics (age, gender, postcode, number of individuals in household)
- The first ‘baseline shopping list’ (i.e. the items selected for purchase prior to the provision of any dietary advice)

At each subsequent visit to the online shopping site the information collected and stored will be:

- The second (third, fourth… etc) ‘baseline shopping lists’ (i.e. the items selected for purchase prior to the provision of dietary advice at subsequent visits) and the second (third, fourth… etc) ‘follow-up shopping list’ (i.e. the items actually purchased after the prompt about dietary advice at subsequent visits).

### PRIMARY OUTCOME

The primary outcome will be the difference between randomised groups in the mean percentage of saturated fat, in the shopping purchased online, during the 6-month follow-up period.

### STATISTICAL ANALYSIS

The analyses will include all randomised participants and will be conducted according to the principle of ‘intention-to-treat’. For the primary outcome, comparisons will be made using unpaired t-tests. Exploration of the effects in participant groups and over time will be explored in an effort to better understand the effects of the intervention, but the emphasis will always be on the overall study result for the primary outcome.

### SAMPLE SIZE ESTIMATES

The sample size of 500 has been selected to provide at least 95% power to detect a 2% or greater absolute difference in the mean saturated fat content of the follow-up shopping lists, between the intervention and control groups (with alpha=0.01). These estimates assume that the mean saturated fat content of the shopping purchased at baseline is between ten and fifteen percent and that the standard deviation about the percentage of saturated fat purchased is 5%. Although online internet shoppers are unlikely to be entirely representative of the general population and are probably more likely to purchase foods with a lower saturated fat content, this is unlikely to move the mean saturated fat content of the shopping list outside the range estimated or to importantly effect the estimate of the standard deviation used. The 2% effect estimate is deemed plausible on the basis of a previous study with similar aims, in which a 3% absolute reduction in purchased saturated fat was achieved using an in-store kiosk to provide computerised dietary advice to customers. The estimate is also compatible with the effects on saturated fat consumption observed in a number of other studies targeting dietary intake rather than food purchases.

### TIMELINE AND MILESTONES

Aug 2002 – Nov 2002 Development and installation of computer software program

Dec 2002 – Jun 2003 500 participants recruited

Dec 2003 End of online intervention and follow-up

Jan 2004 – Jun 2004 Data analysis and presentation of results
