# Supplementary material for: The Effects on Saturated Fat Purchases of Providing Internet Shoppers with Purchase- Specific Dietary Advice: A Randomised Trial
Source: PLoS Clin Trials. 2006 Sep 22;1(5):e22. doi: 10.1371/journal.pctr.0010022 (PMC1574360; doi:10.1371/journal.pctr.0010022)
Supplement: Figure S1 — (58 KB DOC) [file pctr.0010022.sg001.doc]

## Figure S1
